# Microproteins in skeletal muscle: hidden keys in muscle physiology

**DOI:** 10.1002/jcsm.12866

**Published:** 2021-11-30

**Authors:** Bernardo Bonilauri, Bruno Dallagiovanna

**Affiliations:** ^1^ Laboratory of Basic Biology of Stem Cells (LABCET) Carlos Chagas Institute ‐ Fiocruz‐PR Curitiba Paraná Brazil

**Keywords:** Microproteins, ncRNAs, lncRNAs, smORF, Muscle

## Abstract

Recent advances in the transcriptomics, translatomics, and proteomics have led us to the exciting new world of functional endogenous microproteins. These microproteins have a small size and are derived from small open reading frames (smORFs) of RNAs previously annotated as non‐coding (e.g. lncRNAs and circRNAs) as well as from untranslated regions and canonical mRNAs. The presence of these microproteins reveals a much larger translatable portion of the genome, shifting previously defined dogmas and paradigms. These findings affect our view of organisms as a whole, including skeletal muscle tissue. Emerging evidence demonstrates that several smORF‐derived microproteins play crucial roles during muscle development (myogenesis), maintenance, and regeneration, as well as lipid and glucose metabolism and skeletal muscle bioenergetics. These microproteins are also involved in processes including physical activity capacity, cellular stress, and muscular‐related diseases (i.e. myopathy, cachexia, atrophy, and muscle wasting). Given the role of these small proteins as important key regulators of several skeletal muscle processes, there are rich prospects for the discovery of new microproteins and possible therapies using synthetic microproteins.

## Introduction

Open reading frames (ORFs) have been historically defined as sequences of at least 100 codons initiated by an AUG start codon. Small ORFs (smORFs) have been arbitrarily excluded from proteome annotations. However, this definition is being challenged by the development of new molecular biology and bioinformatic tools.^1^, ^2^ The discovery of translated smORFs in the non‐coding transcripts (ncRNAs) and/or intergenic regions and the presence of alternative ORFs in canonical mRNAs that also encode proteins have emerged as an advance in knowledge of gene expression. The use of ribosome profiling and mass spectrometry allowed the identification of hundreds of new micropeptides coded by smORFs.^3^

**Figure 1:**
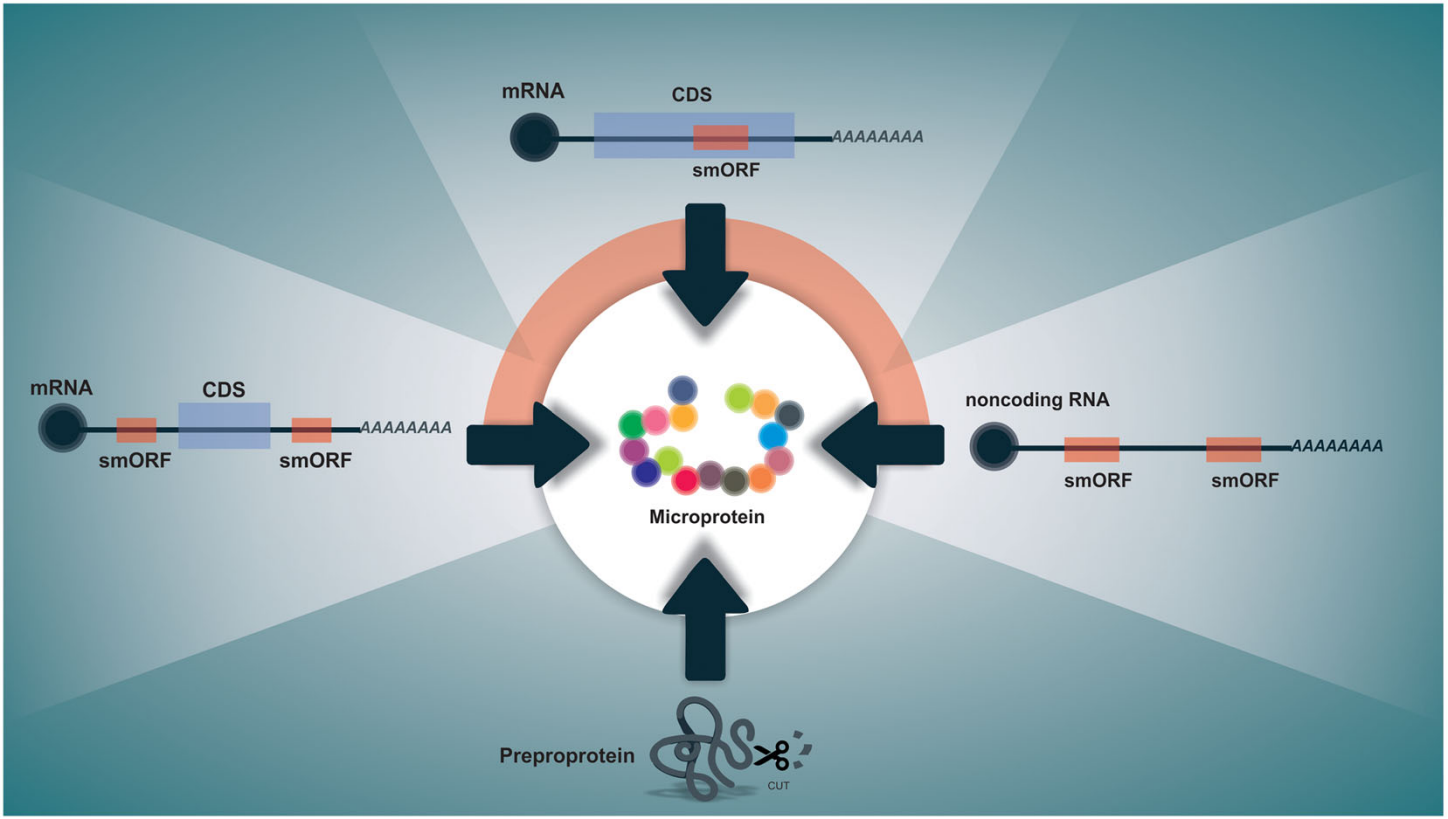
Overview of the origin of microproteins. Translated small ORFs within non‐coding RNAs and canonical mRNAs (overlapping with CDS and UTRs) emerged as major source of microproteins.

Some ncRNAs may thus contain smORFs of up to 100 codons in size that can be translated into functional peptides.^4^, ^5^, ^6^, ^7^ Furthermore, previously designated untranslated regions (UTRs) may have translated smORFs that regulate the translational dynamics of the canonical mRNA and also encode peptides; they are called upstream ORFs (uORFs) in the 5'UTR and downstream ORFs (dORFs) in the 3'UTR.^8^, ^9^, ^10^ Furthermore, translated non‐canonical smORFs have been found to overlap the CDS of mRNAs, encoding microproteins totally different in amino acid composition from the canonical protein. Many of these microproteins are initiated with AUG or non‐AUG start codons, with both in‐frame and out‐of‐frame starts.^11^, ^12^, ^13^, ^14^, ^15^ Together, both lead to an extremely significant increase in the complexity of the regulation of gene expression and the proteome. Interestingly, microproteins derived from smORFs are directly produced by translation, unlike peptide hormones, neuropeptides, and bioactive peptides, which are products of the proteolysis of a preproprotein (e.g. glucagon and insulin)^16^, ^17^, ^18^ (*Figure* 1).

Long non‐coding RNAs (lncRNAs) have aroused particular interest because they contain a large number of smORFs. Initial studies describing lncRNAs date back to the early 1990s.^19^, ^20^, ^21^ These RNAs were characterized as sequences longer than 200 nucleotides (nt), which are unable to encode proteins because they do not contain canonical ORFs.^22^, ^23^, ^24^, ^25^ lncRNAs are involved in a plethora of molecular and cellular processes, including chromatin regulation, transcriptional and post‐transcriptional regulation, splicing, nuclear organization, telomere length, X chromosome inactivation, competing and endogenous RNA (ceRNA), as well as cellular processes such as cell maintenance, development, differentiation, pluripotency, immune response, and cancer.^26^, ^27^, ^28^, ^29^, ^30^ Moreover, lncRNAs have seen clinical use as potential biomarkers for different types of diagnostics due to their tissue/condition‐specific expression.^31^, ^32^, ^33^, ^34^ Although skeletal muscle is a complex tissue, several muscle‐specific lncRNAs appear to regulate muscle development, normal physiology, and diseased states (e.g. cachexia, muscle atrophy, and wasting, and sarcopenia).^35^, ^36^, ^37^, ^38^, ^39^

Accordingly, the search and discovery of smORFs in coding and non‐coding transcripts have grown exponentially in recent years, although only a few smORF‐encoded small proteins have been characterized. Thus, hundreds to thousands of previously non‐annotated smORFs have been identified in genomes ranging from humans to bacteria. However, the identification and characterization of smORF‐derived microproteins is a challenge due to their low expression, the low translational rates of these smORFs in ‘non‐coding’ transcripts, and the non‐identification of the microproteins in complex protein samples due to their low abundance, high turnover, and small size.^16^, ^40^, ^41^ Some computational methods and high‐throughput techniques such as ribosome profiling followed by deep sequencing (Ribo‐seq), transcriptomics (RNA‐seq), and mass spectrometry (proteomics and peptidomics) are therefore being employed to increase the rate of microprotein identification in several cell types and tissues.^14^, ^42^, ^43^, ^44^, ^45^ Even so, few studies have combined these techniques to find smORFs in skeletal muscle tissue, where microproteins have only been identified to date through the manual curation of specific transcripts.^46^, ^47^, ^48^ With the increase of transcriptomics and proteomics studies of the skeletal muscle tissue,^49^, ^50^, ^51^ new perspectives emerge in the field, in order to discover new functional microproteins in this tissue and in different conditions (e.g. comparative studies including young vs. old, athletes vs. non‐athletes, sedentary vs. trained, and healthy vs. disease). Despite technical difficulties and challenges, more microproteins in skeletal muscle are sure to be discovered in the near future.

With this motivation, we seek to provide an overview of the recent discoveries and advances related to this new molecular world, shedding light on the smORF‐derived microproteins with functions in the skeletal muscle system and the implications of the presence or absence of these peptides for muscle physiology.

## Microproteins in muscle: what do we know?

### Microproteins and the sarcoplasmic reticulum

The first studies focusing on the search for animal smORF‐derived microproteins were performed in *Drosophila* and *Zebrafish*, with these proteins exhibiting important functions in cell development and embryogenesis in both organisms.^52^, ^53^, ^54^ Microproteins with functions in the cardiac muscle of flies, mice, and humans have also been discovered.^55^, ^56^ Phospholamban (PLN or PLB), a smORF‐derived microprotein of 52‐amino acid (aa) residues, and the 31‐aa sarcolipin (SLN) are crucial regulators of human cardiac and slow skeletal muscle contraction, interacting with the sarcoplasmic reticulum Ca^2+^ ATPase (SERCA) pump.^57^, ^58^, ^59^ These smORF‐encoded microproteins are encoded in genes previously annotated as CMD1P and MGC12301, respectively (*Table* 1).

**Table 1:** Microproteins in skeletal muscle system

Microprotein	Size (aa)	Gene	Function	Cellular location	Interaction	Ref.
DWORF	34	RP11‐451G4.2	Increase SERCA2a activity in cardiac ventricular muscle by displacing the micropeptide PLN, resulting in an increased calcium cycling and contractility.	Sarcoplasmic reticulum	SERCA pump	47
MLN	46	LINC00948	Inhibition of SERCA1 activity, controlling muscle Ca^2+^ concentration, muscle contraction, and performance.	Sarcoplasmic reticulum	SERCA pump	48
PLN	52	CMD1P	Crucial role in cardiac contractility by inhibition of SERCA2a activity, decreasing the uptake rate of Ca^2+^ into SR.	Sarcoplasmic reticulum	SERCA pump	56
SLN	31	MGC12301	Inhibition of SERCA1 and SERCA2a activity, decreasing the uptake rate of Ca^2+^ into SR.	Sarcoplasmic reticulum	SERCA pump	59
MYOMIXER, MYOMERGER, MINION	84^a^	RP1‐302G2.5	Crucial role in the fusogenic process of myoblasts membrane to promote myotubes (multinucleated myofibres).	Plasma membrane	Myomaker	88, 89, 90
BRAWNIN	71	C12orf73	Regulation of cellular bioenergetics through direct interaction, assembly, and stabilization of the respiratory chain complex III.	Mitochondria (inner membrane)	Respiratory complex III	109
MIEF1, altMiD51	70	MiD51	Functions related to mitochondrial fission process and regulation of mitochondrial translation.	Mitochondria (Matrix)	Drp1, mitoribosome	140
MOTS‐c	16	MT‐RNR1	Regulation of muscle metabolism and glucose metabolism through improving insulin sensitivity and AMPK phosphorylation.	Mitochondria Nucleus	?	134
MTLN, MOXI, LEMP, MPM	56	LINC00116	Regulation of the mitochondrial membrane protein complex assembly, enhance mitochondrial membrane potential, reduce ROS production, and increase basal and maximal respiration rates.	Mitochondria (inner membrane)	Cardiolipin, MTFP	99, 100, 101, 102, 103
PIGBOS	54	RP11‐139H15.1	Modulation of cellular sensitivity to ER stress by regulation of the unfold protein response (UPR) pathway in endoplasmic reticulum.	Mitochondria (outer membrane)	CLCC1	114
SPAR, SPAAR	90	LINC00961	Regulation of the mTORC1‐signalling pathway in response to amino acids stimulation.	Lysosome	v‐ATPase	148
LDANP1	87	USPL1	Regulated triacylglycerol storage and insulin sensitivity in murine myoblasts.	Lipid droplets	?	160
HN	24	MT‐RNR2	Regulation of cellular stress by increasing AMPK phosphorylation and ATP levels, and anti‐apoptotic activity by blocking the translocation of BAX to mitochondria.	Cytosol	BAX, BimEL	119
lncRNA‐Six1‐ORF2	65	lncRNA‐Six1	Regulation *in cis* of Six1 gene, which presents myogenic function in chicken.	Cytosol Nucleus	?	161
SHLP1, SHLP2	24 26	MT‐RNR2	Improve mitochondrial metabolism, reduce ROS and apoptosis, and increase oxygen consumption rate and ATP production.	Cytosol	?	131

^a^ Human microprotein size.

SERCA controls the reuptake of cytosolic Ca^2+^ (i.e. released from the sarcoplasmic reticulum by the ryanodine receptor), promoting muscle relaxation and Ca^2+^ restoration in the sarcoplasmic reticulum (SR). The pump is regulated through interactions with several peptides and post‐translational modifications (e.g. phosphorylation and nitrosylation).^60^, ^61^ The SERCA protein family includes SERCA1, SERCA2 (SERCA2a and SERCA2b), and SERCA3, although SERCA3 is absent from muscle tissue. Meanwhile, SERCA2a is expressed only in slow skeletal muscle and cardiac muscle, while SERCA2b is dominantly expressed in smooth muscle and many nonmuscle cell types. SERCA1 is specifically expressed in slow and fast skeletal muscles.^60^, ^62^, ^63^, ^64^ Thus, the activity of SERCA is not only crucial for healthy cardiac and skeletal muscle but also for a number of related tissues involved in molecular responses to myopathies (e.g. Brody disease) and neurodegenerative diseases (e.g. Alzheimer's and Parkinson's diseases), making SERCA and its microprotein partners an excellent therapeutic target.^65^, ^66^, ^67^, ^68^, ^69^, ^70^

**Figure 2:**
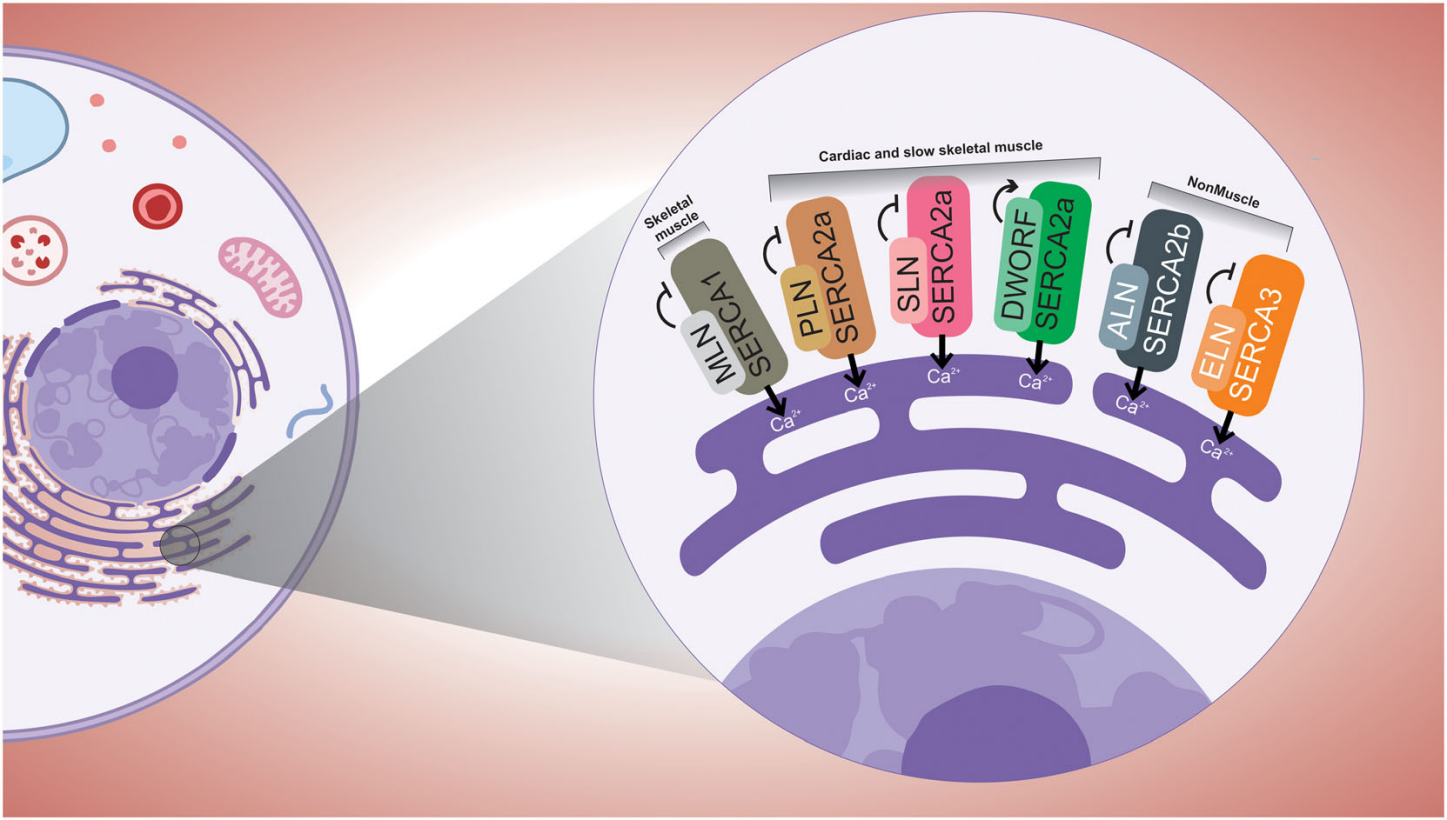
A SERCA‐interacting microproteins family. smORFs‐derived microproteins controlling Ca^2+^ handling in muscle and nonmuscle cells.

Both PLN and SLN directly inhibit the activity of SERCA2a in cardiac muscle, lowering the affinity of the pump for Ca^2+^ and decreasing the uptake rate of Ca^2+^ into the SR, playing a crucial role in cardiac contractility and related diseases^71^ (*Figure* 2). The hearts of PLN‐knockout mice exhibit a significant increase in myocardial contraction resulting from an increase in ventricular relaxation as well as a decreased response to beta‐agonists.^72^, ^73^ Similarly, SLN‐null mice exhibit enhanced relaxation rates in slow/oxidative skeletal muscle, demonstrating the importance of SLN in contractile kinetics, although SLN is poorly expressed in fast skeletal muscle in adult wild‐type mice.^74^ Interestingly, in contrast to mice, human fast skeletal muscle has highly expressed SLN, which affects the regulation of SERCA1 activity.^59^

This evidence on the role of microproteins in controlling cardiac and slow skeletal muscle contraction highlights the need to investigate and discover potential microproteins with the same functions throughout the skeletal muscle system because these microproteins could play crucial roles in the muscle calcium dynamics, and on a wide range of conditions such as muscle development, athletic performance, and degenerative muscle diseases.

The Olson lab therefore used a bioinformatics screening to identify specific uncharacterized skeletal muscle genes, specifically highlighting lncRNA LINC00948. This lncRNA has a smORF of 138 nt that encodes a highly conserved 46‐aa microprotein called Myoregulin (MLN; *Table* 1). MLN is a single transmembrane alpha‐helix microprotein that shares structural similarities with PLN and SLN and also directly interacts with and inhibits SERCA activity.^48^ MLN has specific and robust expression in adult skeletal muscles and directly interacts with SERCA1, unlike PLN and mouse SLN, which directly interact with SERCA2a. This characterizes the action of MLN in the fast‐type skeletal muscle, where its expression is regulated by the transcription factors MyoD and MEF2, which also activate the myogenic programme. Thus, MLN‐knockout mice showed no morphological abnormalities related to muscle and body weight. However, when wild‐type and MLN‐knockout mice are submitted to an exhaustive exercise training (i.e. forced treadmill running), lack of MLN causes a 55% increase in running distance, indicating the important role of MLN in controlling contraction and muscle performance.^48^ Curiously, it was shown that the LIM and cysteine‐rich domains 1 (LMCD1) gene increases skeletal muscle hypertrophy *in vivo*, and their expression levels decrease with aging and disease. This hypertrophic effect is dependent on calcium and calcineurin. Furthermore, LMCD1 and calcineurin together regulate the expression of MLN. When LMCD1 is silenced, levels of MLN increase, and vice versa. Taken together, this indicates that by reducing MLN, LMCD1 de‐represses SERCA activity, leading to an increase in Ca^2+^ influx and decreased fatigue.^75^

Recently, the same lab also discovered the lncRNA LOC100507537/RP11‐451G4.2, which has a smORF that encodes the 34‐aa microprotein dwarf open reading frame (DWORF; *Table* 1). Unlike the PLN, SLN, and MLN microproteins, DWORF enhances SERCA activity in cardiac ventricular muscle by directly interacting with SERCA and displacing the microprotein PLN, resulting in increased calcium cycling and contractility^47^, ^76^, ^77^ (*Figure* 2). Furthermore, the induction of DWORF rescued and attenuated a mouse model of dilated cardiomyopathy (DCM) and increased perfusion pressure in normal and post‐ischaemic reperfusion hearts in rats, indicating a coronary vasoconstrictor action.^78^, ^79^, ^80^ We can therefore speculate the existence of a potential microprotein with similar characteristics and action to DWORF but with specific expression in the skeletal muscle.

Curiously, the lncRNA ZFAS1 was shown to promote intracellular Ca^2+^ overload and contractile dysfunction in a mouse model of myocardial infarction (MI) through direct interaction and inhibition of SERCA2a activity. The expression levels of ZFAS1 are increased in the cytoplasm and SR of MI hearts. This up‐regulation significantly impacts the physiopathology because overexpression of the lncRNA in normal mice triggers deficiencies in cardiac function similar to those seen in MI mice. Moreover, knockdown of ZFAS1 partially reverses the contractile dysfunction related to ischaemia. The lncRNA acts through a dual activity, repressing the expression levels and directly interacting with SERCA2a.^81^ Previously, ZFAS1 was shown to be involved in several molecular mechanisms and in a variety of pathologies (e.g. many cancer types and cardiovascular and neurological conditions). Furthermore, it is associated with ribosomal machinery, which may indicate the presence of a smORF‐encoding peptide.^82^, ^83^, ^84^, ^85^, ^86^

More recently, another two functional microproteins with SERCA‐inhibitory activity were discovered and characterized in nonmuscle cell types: the 56‐aa microprotein endoregulin (ELN) from the SMIM6 gene (previously known as C17orf110) and the 65‐aa microprotein another‐regulin (ALN) from the murine 1810037I17Rik gene.^87^ Together, these results support a previously unappreciated complexity and the need for a greater understanding of microproteins and their functions in the physiology of the skeletal muscle system in both healthy and disease conditions.

### Microprotein and myoblast fusion

A crucial step in muscle development and function is the fusion of myoblasts to form multinucleated myofibres. Recently, the 84‐aa microprotein Myomixer (also known as Minion and Myomerger) was discovered and shown to play a critical role in this fusogenic process (*Table* 1). Myomixer is a conserved microprotein primarily located in the plasma membrane and encoded in a smORF of the gene previously known as RP1‐302G2.5/Gm7325. Its expression is greater during myogenesis and lower after myoblast fusion.^88^, ^89^, ^90^ Interestingly, this small single‐pass membrane protein has orthologues in different species ranging from mammals, reptiles, and amphibians.^91^ More recently was shown a Myomixer orthologue in the lamprey, a jawless ancient vertebrate. Although lamprey Myomixer is a 583‐aa protein and exhibits sequence divergence, it is capable to replace human Myomixer and promote myoblast fusion. No Myomixer orthologue was found in non‐vertebrate chordate groups, indicating that Myomixer is a vertebrate‐specific gene with myoblast fusion functions, conserved from lampreys to humans.^92^

In response to muscle injury (CTX), Myomixer expression is immediately increased and is essential for satellite cell fusion, demonstrating an important function during the process of muscle regeneration. Besides, Myomixer‐knockout mice embryos have lack of skeletal muscle and absence of multinucleated myofibres, which Myomixer being required for skeletal muscle development *in vivo* and *in vitro*.^88^, ^93^ Similar results are also seen during myogenesis and myoblast fusion of zebrafish.^91^

Myomixer acts in association with and activates the plasma membrane fusogenic protein Myomaker, which is dependent on Myomixer for normal myoblast fusion through pore formation. Curiously, for proper and efficient fusion, Myomaker must be expressed in both fusing cells, while Myomixer can be expressed in only one of them, but when expressed in both cells, the fusion efficiency is significantly increased. In particular, complete loss of Myomixer results in fusion defects, although not disrupting all *in vitro* syncytium formation. Most cells lacking Myomixer remain mononuclear (i.e. 63%), albeit the remainder are binucleated myocytes or small myotubes containing three to five myonuclei.^94^ Furthermore, the muscle‐related genes MyoD/MyoG control the expression of both Myomaker and Myomixer, and the loss of MyoD results in the abolishment of myotube formation.^94^ The exact mechanism by which Myomixer acts on the fusogenic process is not entirely known. However, it has been hypothesized that Myomixer shifts the spontaneous curvature of the outer monolayer of the plasma membrane to more positive values, promoting fusion by accelerating the hemifusion‐to‐pore transition.^95^, ^96^, ^97^

### Microproteins and skeletal muscle mitochondria

Mitochondria play a critical role in whole‐body metabolism, and the processes that govern the organelle's dynamics directly affect the physiology of the skeletal muscles. For example, processes that regulate the quantity, of organelles, morphology, biogenesis, and mitophagy, the importation and exportation of proteins and others, are crucial for skeletal muscle function.^98^ Several proteins and peptides participate in this dynamic system, and it has recently been discovered that microproteins also play a key role in mitochondrial homeostasis.

**Figure 3:**
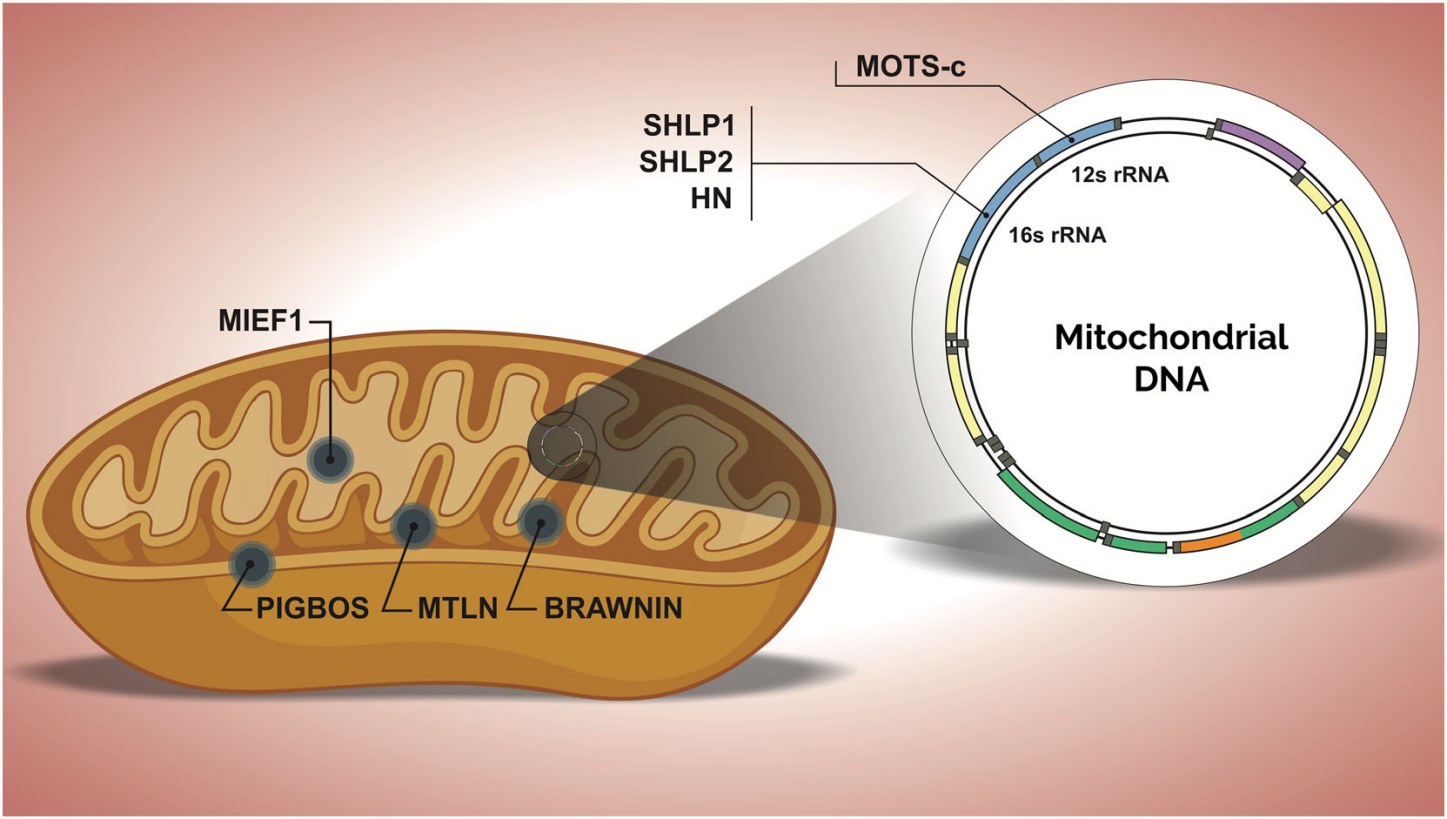
Microproteins and skeletal muscle mitochondria. Microproteins located in different sites of mitochondria show crucial and distinct functions. Mitochondiral DNA (mtDNA) also encoded important microproteins in cellular and mitochondrial dynamics.

One of these microproteins is Mitoregulin (MTLN), also known as microprotein regulator of β‐oxidation (MOXI), lncRNA‐encoded microprotein (LEMP), and microprotein in mitochondria (MPM; *Table* 1). MTLN is derived from a smORF from the lncRNA LINC00116, which is a conserved muscle‐enriched single‐pass transmembrane microprotein with a size of 56 aa.^99^, ^100^, ^101^, ^102^, ^103^ It is located in the inner mitochondrial membrane (IMM) and binds to cardiolipin (CL; a phospholipid that maintains the integrity of the mitochondrial membrane) to regulate the assembly of the protein complex in the membrane (*Figure* 3). MTLN also directly interacts with the mitochondrial trifunctional protein (MTFP), an enzyme embedded in the IMM, which catalyses β‐oxidation of long‐chain fatty acids. Curiously, MTFP acts as specific monolysocardiolipin acyltranseferase (MLCL‐AT) for remodelling cardiolipin, both *in vivo* and *in vitro*.^104^ This remodelling of cardiolipin may be of particular importance for specific processes such as the formation of respiratory supercomplexes and bioenergetics, as well as in mitochondrial architecture and organization.^105^ Furthermore, the expression of cardiolipin is reduced in individuals with cancer, resulting in oxidative phosphorylation dysfunction and increased muscle catabolism (i.e. cachexia), which may indicate a synergistic action of the MTLN–MTFP–Cardiolipin axis in muscle health and disease.^106^

*In vitro* overexpression of MTLN results in increased mitochondrial membrane potential with a concomitant reduction in the production of reactive oxygen species (ROS) and extracellular acidification rates and increased basal and maximal respiration rates and Ca^2+^ retention capacity.^99^ In mice, the expression of MTLN (annotated as 1500011K16Rik) increases during *in vitro* myogenic differentiation, while knockdown of the RNA results in defects in myotube formation and decreased oxygen consumption and ATP production, in addition to reduced expression levels of myosin heavy chain (MHC).^101^, ^102^ MTLN‐knockout mice are born normally but go on to exhibit smaller muscle size and weight, along with a significant reduction in the cross‐section area, lowered fatty acid oxidation capacity, and β‐oxidation. Mice lacking MTLN show muscle weakness and worse muscle performance (e.g. running, swimming, maximum grip force of limbs, and tendency to fall off the rotarod).^100^, ^101^, ^102^

Moreover, MTLN is also expressed in adipose tissue, an important tissue that controls whole‐body metabolism and energy homeostasis. MTLN regulates triglyceride clearance by regulating lipolysis and mitochondrial β‐oxidation in human and murine adipocytes.^107^ Interestingly, MTLN is expressed and translated in undifferentiated human adipose‐derived stem cells and significantly increases after adipogenic induction, a pattern that has also been demonstrated in the adipogenesis of human embryonic stem cells.^107^, ^108^ Taken together, these results indicate that MTLN appears to play a crucial role in myogenic differentiation and muscle physiology, which has a significant impact on mitochondrial respiratory activity and membrane potential, ROS production, β‐oxidation, and ATP production.

Another conserved smORF‐derived microprotein also plays a crucial role in mitochondrial function. The 71‐aa microprotein BRAWNIN is derived from the C12orf73 gene and is also located in IMM, playing an essential role in cellular bioenergetics by directly assembling and stabilizing the respiratory chain complex III^109^ (*Figure* 3). BRAWNIN expression has been detected in mouse, human, and zebrafish cardiac and skeletal muscle tissues. Knockout of BRAWNIN in zebrafish results in normal myogenesis without signs of myopathy and dystrophy, although it does cause significant reductions in the maximum respiration rate and ATP production in skeletal muscle mitochondria, resulting in severe mitochondrial deficiency and growth retardation.^109^ Furthermore, the protein levels of BRAWNIN robustly increase upon AMP‐activated protein kinase (AMPK) induction and are also increased after PGC1‐α overexpression, because PGC‐1α is a master regulator of mitochondrial biogenesis that is also activated by AMPK. This directly implicates the latter in the regulation of skeletal muscle under specific conditions such as physical activity (e.g. endurance and HIIT) and fasting, both of which stimulate the activation of AMPK‐PGC‐1α and, consequently, can increase BRAWNIN levels.^109^, ^110^, ^111^, ^112^, ^113^

Recently, an outer mitochondrial membrane (OMM) microprotein was discovered. The conserved 54‐aa microprotein PIGBOS is encoded from the smORF of the PIGBOS1 gene (previously annotated as RP11‐139H15.1). Surprisingly, it interacts with the endoplasmic reticulum (ER) and plays an important role in the regulation of unfolded protein response (UPR) pathway.^114^ Although the expression of the PIGBOS microprotein has been shown in some tissues (e.g. pancreas, brain, heart, kidney, and liver), little is understood about its expression and role in the skeletal muscle system.^114^ Because skeletal muscle contains an extensive network of SR and the organelle is essential for the regulation of proteostasis, we can speculate that the mitochondrial PIGBOS microprotein has an important role in SR homeostasis (*Figure* 3).

The SR stress‐induced UPR pathway is an essential feature of myogenic differentiation due to the activation of apoptotic pathways (i.e. caspase‐12, caspase‐9, and caspase‐3) for the selective elimination of incompetent and vulnerable myoblasts. Furthermore, the activation of UPR also occurs in the skeletal muscle due to aging, muscular diseases, and physical exercise.^115^, ^116^ The PIGBOS microprotein thus specifically interacts with the ER transmembrane chloride channel CLIC‐like 1 protein (CLCC1) to modulate cellular sensitivity to ER stress. When the UPR pathway is induced, loss of PIGBOS results in increased levels of all UPR target genes. Conversely, the *in vitro* overexpression of PIGBOS results in a modulation of ER stress and the UPR pathway.^114^ Interestingly, the loss of CLCC1 results in the accumulation of unfolded proteins in the ER, neurodegeneration, and muscle wasting and atrophy in a mutant mouse model, with CLCC1 normally expressed in the skeletal muscle of wild‐type mice.^117^ Together, these results imply a possible role for PIGBOS in the homeostasis of skeletal muscle proteins.

Although all of the microproteins mentioned so far are encoded by nuclear‐derived genes, alternative reading frames and smORFs are also present in the mitochondrial genome.^118^ The first mitochondrial‐derived peptide to be identified was the 24‐aa Humanin (HN), which has several cytoprotective and metabolic effects. HN was initially discovered using a cDNA library approach,^119^ encoded by a smORF in the 16S ribosomal RNA (MT‐RNR2 gene). The expression of HN has been demonstrated in several organs, including the skeletal muscle and heart; it is also secreted into blood circulation^120^ (*Figure* 3). Although it is encoded by the mitochondrial genome, HN is located in the cytoplasm and is produced in response to cellular stress, enhancing AMPK phosphorylation and ATP levels, as well as having anti‐apoptotic effects by preventing the translocation of Bcl2‐associated X protein (BAX) from the cytosol to mitochondria, suppressing cytochrome c release and apoptosis.^123,124^ Moreover, HN directly binds to and inhibits the extra‐long isoform of Bim, another proapoptotic protein of the Bcl‐2/Bax family.^125^

Curiously, the expression levels of HN are reduced in the skeletal muscle of chronic kidney disease patients, with this reduction associated with lower mitochondrial density, oxidative stress, and systemic inflammation.^120^ On the other hand, HN has been found to be increased in the skeletal muscle of patients with chronic progressive external ophtalmoplegia (CPEO) and those with mitochondrial encephalomyopathy with lactic acidosis and stroke‐like episodes (MELAS), suggesting that HN expression may be induced in response to defects in energy production, making it an excellent therapeutic candidate for these diseases.^126,127^ Its levels in skeletal muscle are heightened in untrained subjects after a bout of HIIT (acute exercise) and in prediabetic subjects after 12 weeks of resistance training (chronic exercise).^128,129^ Likewise, a MELAS patient who underwent resistance training demonstrated improvements in skeletal muscle and mitochondrial function,^130^ which may indicate a possible action of the HN microprotein.

Similarly, six newly discovered microproteins, called small humanin‐like peptides (SHLP1–6), are encoded by the same 16S ribosomal RNA (MT‐RNR2 gene).^131^ The mouse expression levels of all six microproteins vary in different tissues, with the 24‐aa SHLP1 microprotein detected in several tissues, including the heart and skeletal muscle. The 26‐aa SHLP2 microprotein was also detected in several tissues, with the highest expression recorded in the skeletal muscle and liver (*Figure* 3). Meanwhile, SHLP3–6 (38 aa, 26 aa, 24 aa, and 20 aa, respectively) were mostly detected in other tissues such as the brain, kidney, spleen, and testis. Although little is known about their functions and mechanisms, some clues have emerged. For example, SHLP2 and SHLP3 act similarly to HN, improving mitochondrial metabolism, reducing ROS and apoptosis, and increasing oxygen consumption rate and ATP production.^131^ SHLP2 treatment also affects the concentrations of lipid metabolites and amino acids in the plasma of high‐fat diet fed mice, playing a positive role in regulating metabolic disorders and aging.^131,132^ Furthermore, *in vitro* treatment with SHLP2 of cells affected by age‐related macular degeneration induced anti‐apoptotic effects, restored the levels of oxidative phosphorylation complexes, and increased the number of mtDNA copies.^133^ However, further studies are necessary to better understand the effects of SHLPs on skeletal muscle physiology.

Finally, the 16‐aa MOTS‐c microprotein (mitochondrial ORF of the 12S rRNA‐c) is encoded within the MT‐RNR1 gene and is co‐located in the mitochondria^134^ (*Figure* 3). MOTS‐c is markedly expressed in the skeletal muscle, where expression levels are reduced during aging. In addition, it is expressed in various tissues and released in circulation (i.e. mitokine) in humans and rodents. It directly regulates muscle and glucose metabolism by improving insulin sensitivity (i.e. stimulating glucose uptake) and routing glucose to the pentose phosphate pathway (PPP), which provides carbon for purine biosynthesis. MOTS‐c also inhibits the folate cycle (5Me‐THF), increasing the levels of ZMP (5‐aminoimidazole‐4‐carboxamide ribonucleotide) and consequently enhancing the phosphorylation of AMPK.^134,135^

Surprisingly, under metabolic stress, MOTS‐c translocates to the nucleus and regulates several metabolic genes such as stress‐responsive transcription factors and genes with antioxidant response elements to promote cellular homeostasis, this translocation being AMPK dependent.^136^ One way to induce metabolic stress and/or AMPK activation is through physical exercise, and MOTS‐c levels increased in skeletal muscle after a bout of HIIT, remaining elevated after 4 h of rest. In addition, the treatment of younger and older mice with MOTS‐c for 2 weeks enhances physical capacity and body composition, while treatment for only 7 days improves skeletal muscle insulin sensitivity. A higher dose of MOTS‐c results in 100% of younger mice reaching high speeds on the treadmill (sprint test), with only 17% of the control and low‐dose mice groups obtaining the same result.^137^ Treatment with MOTS‐c also prevents obesity (i.e. reduced fat accumulation) and hyperinsulinaemia, drastically reducing hepatic lipid accumulation and promoting skeletal muscle expression of GLUT4 in high‐fat diet fed mice.^134^ Furthermore, treatment with MOTS‐c results in increased cold tolerance and thermogenic gene expression, in addition to white‐brown fat conversion and brown adipose tissue activation.^138,139^ These results suggest that MOTS‐c plays a direct role in promoting whole‐body metabolism and skeletal muscle adaptations.

As previously mentioned, some smORFs in transcripts with a canonical coding sequence (CDS) are also translated and generate microproteins. This is the case of the gene MID51, which contains a smORF in the 5'UTR (uORF) that encodes the 70‐aa mitochondrial elongation factor 1 (MIEF1, also known as altMiD51) microprotein^140^ (*Table* 1). Canonical MiD51 is a mitochondrial protein (OMM) responsible for the recruitment of the cytosolic Drp1 to mitochondria to promote the fission process.^141,142^ MIEF1 can be robustly detected in mass spectrometry and ribosome profiling assays. Similar to the canonical protein, the microprotein is also located in mitochondria and promotes mitochondrial fission.^140,143^ However, knockout of the MIEF1 microprotein results in a distinct transcriptional pattern compared with knockout of canonical protein, which causes differential expression of mitochondrial fusion and fission genes.^3^ In addition, MIEF1 is located in the mitochondrial matrix and interacts with the mitoribosomes, which are responsible for the translation of several mitochondrial‐encoded mRNAs for the production of respiratory complexes. This directly implies the involvement of MIEF1 in the regulation of mitochondrial translation, having similar or distinct expression pattern in comparison with the canonical protein in different cell lines and conditions.^144,145^

Curiously, no study thus far has focused on the activity of MIEF1 in the skeletal muscle, although MID51 is highly expressed in foetal skeletal muscle, heart, brain, and kidney and is also expressed (to a lesser extent) in adult skeletal muscle, heart, pancreas, and kidney^146,147^ (*Figure* 3). Because skeletal muscle mitochondrion is a highly active organelle with specific dynamics, MIEF1 may play an important role in different stages of muscle development. It may be involved in responses to aging, physical exercise, cellular stress, myopathy, and metabolic and mitochondrial diseases.

### Other skeletal muscle‐related microproteins

The 90‐aa small regulatory polypeptide of amino acid response (known as SPAR or SPAAR) is a microprotein encoded by the lncRNA LINC00961, located in the late endosome and lysosomal membranes (*Table* 1). LINC00961/SPAR is highly expressed in the human heart, skeletal muscle, and lung tissue, with the microprotein playing an important role in muscle regeneration.^148^

SPAR demonstrates specific regulation of the mTORC1‐signalling pathway in response to amino acids. mTORC1 controls protein synthesis and cell growth and is activated through amino acid stimulation and growth factors. This signalling pathway plays a central role in skeletal muscle physiology and is involved in muscle hypertrophy, fibre type specification, and regeneration.^149–151^ Under amino acid stimulation, mTORC1 is activated at the lysosomal surface, a response that is dependent on the v‐ATPase/Ragulator/Rag‐GTPase complexes. The v‐ATPase complex interacts with the Ragulator complex, activating the Rag‐GTPases and consequently activating mTORC1.^152,15^ ^3^ SPAR thus directly interacts with the v‐ATPase complex (ATP6V0A1 and ATP6V0A2 subunits), inhibiting the activation of the v‐ATPase/Ragulator/Rag complexes and mTORC1. When SPAR is overexpressed, mTORC1 is highly inhibited, not being translocated to the lysosomal surface, while knockdown of the microprotein results in mTORC1 activation, although SPAR is regulated as a response to amino acid stimulation and not to growth factor stimulation. Because LINC00961 is highly expressed in human skeletal muscle, the mouse homologue transcript (5430416O09Rik) is also highly expressed. SPAR‐knockout mice exhibit myoblasts and myofibres with intact v‐ATPase functions. However, induction of muscle injury (CTX) in these mice results in an increase in the mTORC1 activation and muscle regeneration, promoting stem cell proliferation, differentiation, and maturation.^148,149^ Taking these results together, SPAR appears to have an important regulatory effect on the mTORC1 pathway in muscle maintenance and regeneration. Despite this, LINC00961 may have a dual function, acting as a coding RNA and a regulatory RNA. As seen in endothelial cells, where the lncRNA increases anti‐angiogenic activity, and the microprotein exhibits pro‐angiogenic activity. This lncRNA/microprotein combination may therefore perform specific functions in different cells, tissues, and conditions (e.g. cancer and cardiac diseases).^154–159^

More recently, the 87‐aa LDANP1 microprotein from an out‐of‐frame smORF of the USPL1 gene was discovered in the lipid droplets (LD) of murine myoblasts (*Table* 1). Overexpression of the microprotein reduces total triacylglycerol levels in C2C12 cells undergoing incubation with oleate (OA) and enhances insulin sensitivity, although its exact mechanism of action is still unknown. Curiously, LDANP1 was only detected after sample enrichment by immunoprecipitation and cannot be detected in whole cell lysate.^160^ Therefore, smORF‐derived microproteins located in the LD may directly act in the regulation of lipid and glucose metabolism in the skeletal muscle, thus playing an important role in metabolic diseases such as diabetes, obesity, and cachexia.

Ultimately, despite some lncRNAs not having evolutionary conservation and demonstrating species specificity, they show molecular similarities, such as the presence of smORFs and regulatory functions. For example, the chicken lncRNA‐Six1 is highly expressed in breast muscle and acts as ceRNA, sponging miR‐1611 to regulate Six1 protein expression and skeletal muscle fibre type specification, myoblast proliferation, and differentiation, in addition to encoding a 65‐aa microprotein (*Table* 1). Overexpression of lncRNA‐Six1 microprotein promotes migration and cell proliferation, with the microprotein possibly acting through regulation of Six1 *in cis*, which is crucial for myogenesis.^161,162^

## Potential microproteins in skeletal muscle: different tissues, same functions?

Some newly discovered smORF‐derived microproteins may have specific functions in muscle tissue, although these have not yet been demonstrated or have only been demonstrated in other cell and tissue types. We can thus hypothesize some possible mechanisms for further studies in the field.

For example, the lncRNA MIR155HG is the host gene of microRNA‐155 and encodes the 17‐aa microprotein P155 (also known as miPEP155).^163^ Interestingly, miR‐155 is involved in immune response, and its presence is increased in activated B cells, macrophages, and dendritic cells^.164^ Furthermore, the role of miR‐155 in skeletal muscle is poorly studied. Their expression level is higher in aged satellite cells (SC), where they also demonstrate an enhanced expression of the differentiation genes (i.e. myoblast commitment) and not the SC markers Pax7 and Notch1. Because the expression of the miR‐155 is associated with inflammation, their baseline expression level is extremely low in normal muscle cells. However, it increases markedly on the first day after muscle injury in mice, decreasing gradually to the baseline level by day nine.^165,166^ Corroborating these findings, miR‐155‐knockout mice have delayed muscle regeneration after injury (CTX), with the new muscle fibres smaller in size. However, the effects of miR‐155 may be related to the balance of pro‐inflammatory and anti‐inflammatory macrophages during muscle regeneration.^166^ On the other hand, P155 microprotein was first found in human and mouse dendritic cells, where it modulates lysosomal localization of antigens and the MHC‐II antigen presentation to T cells (CD4^+^). P155 acts by binding to the HSC70 chaperone, probably impairing the HSC70 function as an antigen transporter in dendritic cells. It also suppresses the expression of MHC‐II, demonstrating an inflammatory suppression function (i.e. anti‐inflammatory response). Curiously, HSC70 is involved in the assembly of myosin intermediates in myoblasts but not in the mature myofibres.^167^ An uncontrolled and persistent inflammatory response in skeletal muscle may thus impair regenerative capacity and result in muscle atrophy, making MIR155HG products a potential target for muscle regeneration. Thus, the microRNA and microprotein appear to act in opposite directions, with the former acting in a pro‐inflammatory manner and the latter acting as an inflammatory suppressor.^168,163,169^

This pro‐inflammatory environment in muscle biology demonstrates several beneficial physiological effects on muscle homeostasis. Interleukin‐6 (IL6), associated with the JNK/STAT3 signalling pathway, represents important activators of inflammatory processes and muscle regeneration. However, chronic hyperactivation of IL6 and STAT3 is involved in muscle wasting, atrophy, sarcopenia, and cachexia.^170–172^ A 60‐aa microprotein derived from the lncRNA LINC00908 was recently discovered, named ASRPS (a small regulatory peptide of STAT3). This microprotein directly interacts with STAT3, reducing its phosphorylation, and is down‐regulated in triple‐negative breast cancer (TNBC). A low expression level of ASRPS is associated with poor patient survival. The inactivation of STAT3 activity by ASRPS in TNBC results in reduced tumour angiogenesis and tumour growth.^173^ These results demonstrate the potential therapeutic value of microproteins in diseases such as cancer, while their future use in skeletal muscle conditions and diseases remains a promising avenue for future research.

## Future perspectives

Although there are some technical limitations for the discovery of new microproteins, new studies have demonstrated the real presence and functions of smORF‐derived microproteins, many of which are crucial for certain cellular mechanisms. New perspectives have thus emerged in relation to the skeletal muscle system, necessitating the use of several techniques such as next‐generation sequencing of the total, polysomal, and ribosome‐protected fragments (i.e. RNA‐seq, Poly‐seq, and Ribo‐seq, respectively), mass spectrometry (i.e. proteomics and peptidomics), and functional biochemical and genetics assays (e.g. CRISPR mutagenesis) in muscle cells and in different conditions. The complementarity between these techniques is important due to the difficulty in identifying microproteins in complex proteomic samples, their low abundance and size, turnover rates, challenges in microprotein stabilization and degradation, and the detection of endogenous peptides, microprotein–protein interactions, smORFs that are out‐of‐frame or have non‐AUG start codons, regulatory smORFs that regulate translation, smORFs as sources of antigenic peptides, and others.^3^, ^14^, ^43^, ^48^

These smORF‐derived microproteins have emerged as important elements in skeletal muscle physiology. The effects of these endogenous peptides are crucial in muscle development and regeneration, physiological conditions (e.g. physical exercise and temperature), and muscle diseases. Beyond this, peptide drugs hold great promise for the treatment of different diseases, including myopathies. The synthetic P155 microprotein, when delivered intravenously, had therapeutic effects on autoinflammatory conditions in mice.^163^ Similarly, the administration of recombinant MP31, a newly discovered microprotein derived from a uORF of the PTEN (phosphate and tensin homologue) mRNA, results in the inhibition of glioblastoma xenografts in mice. The MP31 microprotein is located in mitochondria and acts by limiting lactate‐pyruvate conversion. Loss of MP31 increases global lactate metabolism and enhances oxidative phosphorylation.^180^ Interestingly, PTEN exhibits important functions in skeletal muscle development and physiology, principally in the maintenance of muscle satellite cells, although the expression and effects of MP31 are unknown in this tissue.^18T1–183^ Taken together, the mechanisms and functions of the smORF‐derived microproteins under normal conditions and muscle diseases and the use of synthetic microproteins for therapeutic treatment are promising areas for future research related to skeletal muscles.

## Conclusions

Undoubtedly, with all the findings shown here, we can conclude that smORF‐derived microproteins encoded from transcripts and/or regions previously documented as non‐coding play key roles during muscle development (myogenesis), muscle maintenance, regeneration, lipid and glucose metabolism, and skeletal muscle bioenergetics, as well as in different conditions such as physical activity, cellular stress, and muscular‐related diseases (i.e. myopathy, cachexia, atrophy, and muscle wasting). Therefore, it is necessary to search for new microproteins in the skeletal muscle and to promote future therapies using synthetic microproteins in muscle diseases.

## Conflict of interest

The authors declare no conflict of interest.

## Funding

This work was funded by CNPq PROEP/ICC Grant 442353/2019‐7. B.D. received a fellowship from CNPq and B.B. from Fiocruz.

## Supporting information


**Data S1.** Additional references (refs. 121‐184).
