# Supplementary material for: Microproteins in skeletal muscle: hidden keys in muscle physiology
Source: J Cachexia Sarcopenia Muscle. 2021 Nov 30;13(1):100–13. doi: 10.1002/jcsm.12866 (PMC8818594; doi:10.1002/jcsm.12866)
Supplement: Supplementary file 1 — Data S1. Additional references (refs. 121‐184). [file JCSM-13-100-s001.docx]

**REFERENCES**

121. Charununtakorn ST, Shinlapawittayatorn K, Chattipakorn SC, Chattipakorn N. Potential Roles of Humanin on Apoptosis in the Heart. *Cardiovasc Ther* 2016;**34**:107–114.

122. Yen K, Wan J, Mehta HH, Miller B, Christensen A, Levine ME *et al.* Humanin Prevents Age-Related Cognitive Decline in Mice and is Associated with Improved Cognitive Age in Humans. *Sci Rep* 2018;**8**:14212.

123. Guo B, Zhai D, Cabezas E, Welsh K, Nouraini S, Satterthwait AC *et al.* Humanin peptide suppresses apoptosis by interfering with Bax activation. *Nature* 2003;**423**:456–461.

124. Kwon C, Sun JL, Jeong JH, Jung TW. Humanin attenuates palmitate-induced hepatic lipid accumulation and insulin resistance via AMPK-mediated suppression of the mTOR pathway. *Biochem Biophys Res Commun* 2020;**526**:539–545.

125. Luciano F, Zhai D, Zhu X, Bailly-Maitre B, Ricci J-E, Satterthwait AC *et al.* Cytoprotective peptide humanin binds and inhibits proapoptotic Bcl-2/Bax family protein BimEL. *J Biol Chem* 2005;**280**:15825–15835.

126. Kin T, Sugie K, Hirano M, Goto Y-I, Nishino I, Ueno S. Humanin expression in skeletal muscles of patients with chronic progressive external ophthalmoplegia. *J Hum Genet* 2006;**51**:555–558.

127. Kariya S, Hirano M, Furiya Y, Sugie K, Ueno S. Humanin detected in skeletal muscles of MELAS patients: a possible new therapeutic agent. *Acta Neuropathol* 2005;**109**:367–372.

128. Woodhead JST, D’Souza RF, Hedges CP, Wan J, Berridge MV, Cameron-Smith D *et al.* High-intensity interval exercise increases humanin, a mitochondrial encoded peptide, in the plasma and muscle of men. *J Appl Physiol (1985)* 2020;**128**:1346–1354.

129. Gidlund E-K, von Walden F, Venojärvi M, Risérus U, Heinonen OJ, Norrbom J *et al.* Humanin skeletal muscle protein levels increase after resistance training in men with impaired glucose metabolism. *Physiol Rep* 2016;**4**.

130. Venturelli M, Villa F, Ruzzante F, Tarperi C, Rudi D, Milanese C *et al.* Neuromuscular and Muscle Metabolic Functions in MELAS Before and After Resistance Training: A Case Study. *Front Physiol* 2019;**10**:503.

131. Cobb LJ, Lee C, Xiao J, Yen K, Wong RG, Nakamura HK *et al.* Naturally occurring mitochondrial-derived peptides are age-dependent regulators of apoptosis, insulin sensitivity, and inflammatory markers. *Aging (Albany NY)* 2016;**8**:796–809.

132. Mehta HH, Xiao J, Ramirez R, Miller B, Kim S-J, Cohen P *et al.* Metabolomic profile of diet-induced obesity mice in response to humanin and small humanin-like peptide 2 treatment. *Metabolomics* 2019;**15**:88.

133. Nashine S, Cohen P, Nesburn AB, Kuppermann BD, Kenney MC. Characterizing the protective effects of SHLP2, a mitochondrial-derived peptide, in macular degeneration. *Sci Rep* 2018;**8**:15175.

134. Lee C, Zeng J, Drew BG, Sallam T, Martin-Montalvo A, Wan J *et al.* The mitochondrial-derived peptide MOTS-c promotes metabolic homeostasis and reduces obesity and insulin resistance. *Cell Metab* 2015;**21**:443–454.

135. Lee C, Kim KH, Cohen P. MOTS-c: A novel mitochondrial-derived peptide regulating muscle and fat metabolism. *Free Radic Biol Med* 2016;**100**:182–187.

136. Kim KH, Son JM, Benayoun BA, Lee C. The Mitochondrial-Encoded Peptide MOTS-c Translocates to the Nucleus to Regulate Nuclear Gene Expression in Response to Metabolic Stress. *Cell Metab* 2018;**28**:516-524.e7.

137. Reynolds JC, Lai RW, Woodhead JST, Joly JH, Mitchell CJ, Cameron-Smith D *et al.* MOTS-c is an exercise-induced mitochondrial-encoded regulator of age-dependent physical decline and muscle homeostasis. *Nat Commun* 2021;**12**:470.

138. Lu H, Tang S, Xue C, Liu Y, Wang J, Zhang W *et al.* Mitochondrial-Derived Peptide MOTS-c Increases Adipose Thermogenic Activation to Promote Cold Adaptation. *Int J Mol Sci* 2019;**20**.

139. Lu H, Wei M, Zhai Y, Li Q, Ye Z, Wang L *et al.* MOTS-c peptide regulates adipose homeostasis to prevent ovariectomy-induced metabolic dysfunction. *J Mol Med (Berl)* 2019;**97**:473–485.

140. Samandi S, Roy AV, Delcourt V, Lucier J-F, Gagnon J, Beaudoin MC *et al.* Deep transcriptome annotation enables the discovery and functional characterization of cryptic small proteins. *Elife* 2017;**6**.

141. Losón OC, Song Z, Chen H, Chan DC. Fis1, Mff, MiD49, and MiD51 mediate Drp1 recruitment in mitochondrial fission. *Mol Biol Cell* 2013;**24**:659–667.

142. Ma J, Zhai Y, Chen M, Zhang K, Chen Q, Pang X *et al.* New interfaces on MiD51 for Drp1 recruitment and regulation. *PLoS One* 2019;**14**:e0211459.

143. Andreev DE, O’Connor PBF, Fahey C, Kenny EM, Terenin IM, Dmitriev SE *et al.* Translation of 5’ leaders is pervasive in genes resistant to eIF2 repression. *Elife* 2015;**4**:e03971.

144. Rathore A, Chu Q, Tan D, Martinez TF, Donaldson CJ, Diedrich JK *et al.* MIEF1 Microprotein Regulates Mitochondrial Translation. *Biochemistry* 2018;**57**:5564–5575.

145. Delcourt V, Brunelle M, Roy AV, Jacques J-F, Salzet M, Fournier I *et al.* The Protein Coded by a Short Open Reading Frame, Not by the Annotated Coding Sequence, Is the Main Gene Product of the Dual-Coding Gene MIEF1. *Mol Cell Proteomics* 2018;**17**:2402–2411.

146. Liu T, Yu R, Jin S-B, Han L, Lendahl U, Zhao J *et al.* The mitochondrial elongation factors MIEF1 and MIEF2 exert partially distinct functions in mitochondrial dynamics. *Exp Cell Res* 2013;**319**:2893–2904.

147. Zhao J, Liu T, Jin S, Wang X, Qu M, Uhlén P *et al.* Human MIEF1 recruits Drp1 to mitochondrial outer membranes and promotes mitochondrial fusion rather than fission. *EMBO J* 2011;**30**:2762–2778.

148. Matsumoto A, Pasut A, Matsumoto M, Yamashita R, Fung J, Monteleone E *et al.* mTORC1 and muscle regeneration are regulated by the LINC00961-encoded SPAR polypeptide. *Nature* 2017;**541**:228–232.

149. Matsumoto A, Clohessy JG, Pandolfi PP. SPAR, a lncRNA encoded mTORC1 inhibitor. *Cell Cycle* 2017;**16**:815–816.

150. Goodman CA. The role of mTORC1 in regulating protein synthesis and skeletal muscle mass in response to various mechanical stimuli. *Rev Physiol Biochem Pharmacol* 2014;**166**:43–95.

151. Baraldo M, Geremia A, Pirazzini M, Nogara L, Solagna F, Türk C *et al.* Skeletal muscle mTORC1 regulates neuromuscular junction stability. *J Cachexia Sarcopenia Muscle* 2020;**11**:208–225.

152. Zoncu R, Bar-Peled L, Efeyan A, Wang S, Sancak Y, Sabatini DM. mTORC1 senses lysosomal amino acids through an inside-out mechanism that requires the vacuolar H(+)-ATPase. *Science* 2011;**334**:678–683.

153. Bar-Peled L, Sabatini DM. Regulation of mTORC1 by amino acids. *Trends Cell Biol* 2014;**24**:400–406.

154. Spencer HL, Sanders R, Boulberdaa M, Meloni M, Cochrane A, Spiroski A-M *et al.* The LINC00961 transcript and its encoded micropeptide, small regulatory polypeptide of amino acid response, regulate endothelial cell function. *Cardiovasc Res* 2020;**116**:1981–1994.

155. Wu C-T, Liu S, Tang M. Downregulation of linc00961 contributes to promote proliferation and inhibit apoptosis of vascular smooth muscle cell by sponging miR-367 in patients with coronary heart disease. *Eur Rev Med Pharmacol Sci* 2019;**23**:8540–8550.

156. Spiroski A-M, Sanders R, Meloni M, McCracken IR, Thomson A, Brittan M *et al.* The Influence of the LINC00961/SPAAR Locus Loss on Murine Development, Myocardial Dynamics, and Cardiac Response to Myocardial Infarction. *Int J Mol Sci* 2021;**22**.

157. Mu X, Mou K-H, Ge R, Han D, Zhou Y, Wang L-J. Linc00961 inhibits the proliferation and invasion of skin melanoma by targeting the miR‑367/PTEN axis. *Int J Oncol* 2019;**55**:708–720.

158. Chen D, Zhu M, Su H, Chen J, Xu X, Cao C. LINC00961 restrains cancer progression via modulating epithelial-mesenchymal transition in renal cell carcinoma. *J Cell Physiol* 2019;**234**:7257–7265.

159. Liu S, He Y, Shi J, Liu L, Ma H, He L *et al.* STAT1-avtiviated LINC00961 regulates myocardial infarction by the PI3K/AKT/GSK3β signaling pathway. *J Cell Biochem* 2019;**120**:13226–13236.

160. Huang T, Bamigbade AT, Xu S, Deng Y, Xie K, Ogunsade OO *et al.* Identification of Functional Noncoding RNA-encoded Proteins on Lipid Droplets. Cell Biology; 2020 doi:10.1101/2020.04.10.036160.

161. Cai B, Li Z, Ma M, Wang Z, Han P, Abdalla BA *et al.* LncRNA-Six1 Encodes a Micropeptide to Activate Six1 in Cis and Is Involved in Cell Proliferation and Muscle Growth. *Front Physiol* 2017;**8**:230.

162. Ma M, Cai B, Jiang L, Abdalla BA, Li Z, Nie Q *et al.* lncRNA-Six1 Is a Target of miR-1611 that Functions as a ceRNA to Regulate Six1 Protein Expression and Fiber Type Switching in Chicken Myogenesis. *Cells* 2018;**7**.

163. Niu L, Lou F, Sun Y, Sun L, Cai X, Liu Z *et al.* A micropeptide encoded by lncRNA MIR155HG suppresses autoimmune inflammation via modulating antigen presentation. *Sci Adv* 2020;**6**:eaaz2059.

164. Eis PS, Tam W, Sun L, Chadburn A, Li Z, Gomez MF *et al.* Accumulation of miR-155 and BIC RNA in human B cell lymphomas. *Proc Natl Acad Sci U S A* 2005;**102**:3627–3632.

165. Onodera Y, Teramura T, Takehara T, Itokazu M, Mori T, Fukuda K. Inflammation-associated miR-155 activates differentiation of muscular satellite cells. *PLoS ONE* 2018;**13**:e0204860.

166. Nie M, Liu J, Yang Q, Seok HY, Hu X, Deng Z-L *et al.* MicroRNA-155 facilitates skeletal muscle regeneration by balancing pro- and anti-inflammatory macrophages. *Cell Death Dis* 2016;**7**:e2261.

167. Srikakulam R, Winkelmann DA. Chaperone-mediated folding and assembly of myosin in striated muscle. *Journal of Cell Science* 2004;**117**:641–652.

168. Howard EE, Pasiakos SM, Blesso CN, Fussell MA, Rodriguez NR. Divergent Roles of Inflammation in Skeletal Muscle Recovery From Injury. *Front Physiol* 2020;**11**:87.

169. Prel A, Dozier C, Combier J-P, Plaza S, Besson A. Evidence That Regulation of Pri-miRNA/miRNA Expression Is Not a General Rule of miPEPs Function in Humans. *Int J Mol Sci* 2021;**22**.

170. Muñoz-Cánoves P, Scheele C, Pedersen BK, Serrano AL. Interleukin-6 myokine signaling in skeletal muscle: a double-edged sword? *FEBS J* 2013;**280**:4131–4148.

171. Guadagnin E, Mázala D, Chen Y-W. STAT3 in Skeletal Muscle Function and Disorders. *Int J Mol Sci* 2018;**19**.

172. Johnson DE, O’Keefe RA, Grandis JR. Targeting the IL-6/JAK/STAT3 signalling axis in cancer. *Nat Rev Clin Oncol* 2018;**15**:234–248.

173. Wang Y, Wu S, Zhu X, Zhang L, Deng J, Li F *et al.* LncRNA-encoded polypeptide ASRPS inhibits triple-negative breast cancer angiogenesis. *The Journal of Experimental Medicine* 2020;**217**:e20190950.

174. Dallagiovanna B, Pereira IT, Origa-Alves AC, Shigunov P, Naya H, Spangenberg L. lncRNAs are associated with polysomes during adipose-derived stem cell differentiation. *Gene* 2017;**610**:103–111.

175. Marcon BH, Spangenberg L, Bonilauri B, Robert AW, Angulski ABB, Cabo GC *et al.* Data describing the experimental design and quality control of RNA-Seq of human adipose-derived stem cells undergoing early adipogenesis and osteogenesis. *Data in Brief* 2020;**28**:105053.

176. van Heesch S, van Iterson M, Jacobi J, Boymans S, Essers PB, de Bruijn E *et al.* Extensive localization of long noncoding RNAs to the cytosol and mono- and polyribosomal complexes. *Genome Biol* 2014;**15**:R6.

177. Schlesinger D, Elsässer SJ. Revisiting sORFs: overcoming challenges to identify and characterize functional microproteins. *FEBS J* 2021;febs.15769.

178. Prensner JR, Enache OM, Luria V, Krug K, Clauser KR, Dempster JM *et al.* Noncanonical open reading frames encode functional proteins essential for cancer cell survival. *Nat Biotechnol* 2021 doi:10.1038/s41587-020-00806-2.

179. Martinez TF, Chu Q, Donaldson C, Tan D, Shokhirev MN, Saghatelian A. Accurate annotation of human protein-coding small open reading frames. *Nat Chem Biol* 2020;**16**:458–468.

180. Huang N, Li F, Zhang M, Zhou H, Chen Z, Ma X *et al.* An Upstream Open Reading Frame in Phosphatase and Tensin Homolog Encodes a Circuit Breaker of Lactate Metabolism. *Cell Metab* 2021;**33**:128-144.e9.

181. Yue F, Bi P, Wang C, Li J, Liu X, Kuang S. Conditional Loss of Pten in Myogenic Progenitors Leads to Postnatal Skeletal Muscle Hypertrophy but Age-Dependent Exhaustion of Satellite Cells. *Cell Rep* 2016;**17**:2340–2353.

182. Yue F, Bi P, Wang C, Shan T, Nie Y, Ratliff TL *et al.* Pten is necessary for the quiescence and maintenance of adult muscle stem cells. *Nat Commun* 2017;**8**:14328.

183. Shan T, Liu J, Xu Z, Wang Y. Roles of phosphatase and tensin homolog in skeletal muscle. *J Cell Physiol* 2019;**234**:3192–3196.

184. Haehling S, Morley JE, Coats AJS, Anker SD. Ethical guidelines for publishing in the *Journal of Cachexia, Sarcopenia and Muscle* : update 2019. *Journal of Cachexia, Sarcopenia and Muscle* 2019;**10**:1143–1145.
